# *Skeletal Muscle *- one year on

**DOI:** 10.1186/2044-5040-2-1

**Published:** 2012-01-05

**Authors:** David Glass, Kevin Campbell, Michael Rudnicki

**Affiliations:** 1 Novartis Institutes for Biomedical Research, 200 Technology Square, 02139 Cambridge, MA, USA; 2 Howard Hughes Medical Institute, Carver College of Medicine, University of Iowa, Iowa City, IA, USA; 3 Ottawa Hospital Research Institute and University of Ottawa, 725 Parkdale Avenue, K1Y 4E9 Ottawa, ON, Canada

## 

The beginning of 2012 marks the first anniversary of *Skeletal Muscle *[1], and an occasion to thank the scientific community for your support of this new journal.

In its first year, the journal had excellent support, publishing 20 original articles and 16 reviews. We are quite grateful to the expert reviewers who gave their time to increase the quality of these papers - and are proud that all of the submissions received reviews from truly the top experts in our field, almost in every case members of our Editorial Board.

In addition, in August, *Skeletal Muscle *began to be indexed by PubMed [2], increasing the visibility and providing the last step in the "birth" of the journal.

Going forward in 2012 we do hope you will submit your findings to the journal. As you can imagine, Impact Factor is only achieved by first adopters who are willing to support the journal early, before a factor is established. Further, your letters to the journal and citation of journal articles will also help to increase *Skeletal Muscle*'s visibility, which will then in turn improve its Impact Factor - which will help every author who publishes in the journal.

The bottom line is that this is truly a group endeavour; *Skeletal Muscle *is here for the scientist interested in this dynamic tissue. Like its namesake, it can only gather strength with use and exercise - perhaps you can start by including us in your New Year's Resolution for 2012... to help grow the journal by your support.

Thanks and best of luck with your research and education this year!
